# Quinine in Cholera Infantum

**Published:** 1853-09

**Authors:** G. W. Booth

**Affiliations:** Carrollsville, Miss.


From the Southern Med. and Surg. Journal.

*Quinine in Cholera Infantum.* By G. W. Booth, M.D., of Carrollsville, Miss.

As the season is near at hand when that scourge of infancy, Cholera Infantum, usually makes its appearance, I will again invite the attention of the profession to the views I entertain of its etiology and treatment as published in some of the medical journals in 1851. In the communication referred to, I stated it as my opinion that it was of malarial origin. There are many reasons that I could give to sustain the correctness of this opinion, but I deem it unnecessary at this time.

My views respecting the origin of the disease influenced me to use the great and approved antiperiodic, Quinine, in its treatment. The success I met with in combating the disease with this article still farther confirmed me in my conviction as to its predisposing cause. I earnestly solicit the profession to give Quinine a fair trial in Cholera Infantum; I feel confident that no one will regret the experiment. I use other articles to meet particular indications, such as acetate of lead, and calomel, in small doses, for controlling the discharge from the bowels, acting on the liver, &c.

